# Right-to-left shunt with hypoxemia in pulmonary hypertension

**DOI:** 10.1186/1471-2261-9-15

**Published:** 2009-03-31

**Authors:** Jean-Frédéric Vodoz, Vincent Cottin, Jean-Charles Glérant, Geneviève Derumeaux, Chahéra Khouatra, Anne-Sophie Blanchet, Bénédicte Mastroïanni, Jean-Yves Bayle, Jean-François Mornex, Jean-François Cordier

**Affiliations:** 1 Service de pneumologie et centre de référence des maladies orphelines pulmonaires, Hôpital Louis Pradel, Hospices Civils de Lyon, Lyon, France; 2 Université Lyon I, Université de Lyon, Lyon, France; 3 Laboratoire d'exploration fonctionnelle respiratoire, Hôpital Louis Pradel, Hospices Civils de Lyon, Lyon, France; 4 Laboratoire d'échocardiographie, Hôpital Louis Pradel, Hospices Civils de Lyon, Lyon, France; 5 UMR754 RPC, Ecole Nationale Vétérinaire de Lyon, Ecole pratique des hautes études, IFR128, INRA, Lyon, France

## Abstract

**Background:**

Hypoxemia is common in pulmonary hypertension (PH) and may be partly related to ventilation/perfusion mismatch, low diffusion capacity, low cardiac output, and/or right-to-left (RL) shunting.

**Methods:**

To determine whether true RL shunting causing hypoxemia is caused by intracardiac shunting, as classically considered, a retrospective single center study was conducted in consecutive patients with precapillary PH, with hypoxemia at rest (PaO_2 _< 10 kPa), shunt fraction (Qs/Qt) greater than 5%, elevated alveolar-arterial difference of PO_2 _(AaPO_2_), and with transthoracic contrast echocardiography performed within 3 months.

**Results:**

Among 263 patients with precapillary PH, 34 patients were included: pulmonary arterial hypertension, 21%; PH associated with lung disease, 47% (chronic obstructive pulmonary disease, 23%; interstitial lung disease, 9%; other, 15%); chronic thromboembolic PH, 26%; miscellaneous causes, 6%. Mean pulmonary artery pressure, cardiac index, and pulmonary vascular resistance were 45.8 ± 10.8 mmHg, 2.2 ± 0.6 L/min/m^2^, and 469 ± 275 dyn.s.cm^-5^, respectively. PaO_2 _in room air was 6.8 ± 1.3 kPa. Qs/Qt was 10.2 ± 4.2%. AaPO_2 _under 100% oxygen was 32.5 ± 12.4 kPa. Positive contrast was present at transthoracic contrast echocardiography in 6/34 (18%) of patients, including only 4/34 (12%) with intracardiac RL shunting. Qs/Qt did not correlate with hemodynamic parameters. Patients' characteristics did not differ according to the result of contrast echocardiography.

**Conclusion:**

When present in patients with precapillary PH, RL shunting is usually not related to reopening of patent *foramen ovale*, whatever the etiology of PH.

## Background

Pulmonary hypertension (PH) is characterized by increased pulmonary artery pressure, ultimately leading to right heart failure and death. PH encompasses various etiologic groups, including pulmonary arterial hypertension (PAH) – group 1, PH related to left heart disease – group 2, PH associated with lung diseases and/or hypoxemia – group 3, PH due to chronic thrombotic and/or embolic disease – group 4, and miscellaneous disorders, including sarcoidosis and Langerhans cell histiocytosis – group 5 [1].

PAH *per se *affects small pulmonary arteries, with vascular remodelling leading to progressive increase in pulmonary vascular resistance (PVR) [2]. It may be idiopathic, familial, or associated with a variety of conditions merged under the common denomination of PAH due to similarities in histopathological features, natural history, and treatment [1]. Precapillary PH is defined by PH with pulmonary artery wedge pressure (PAWP) no greater than 15 mmHg and PVR greater than 240 dyn.s.cm^-5 ^[1]. In addition to PAH, precapillary PH may be encountered as a result of various disorders (etiologic groups 3, 4, and 5), and may also be characterized by remodelling of the small pulmonary arteries although less data are available than in PAH.

Mild to moderate hypoxemia is common in precapillary PH, and most often coexists with respiratory alkalosis. Hence, the mean PaO_2 _was 9.5 ± 2 kPa in the National Institutes of Health PAH registry [3] and 9.2 ± 1.9 kPa in a more recent study [4]. However, hypoxemia in PH may sometimes be severe and contribute to exercise intolerance. Desaturation of 10% or more during 6-minute walk test is associated with a relative mortality risk of 2.9 in PAH [5]. Desaturation may occur overnight in as many as 60% of patients with PAH [6].

Hypoxemia in precapillary PH may possibly be related to ventilation/perfusion mismatch [7,8], low diffusion capacity, low mixed venous PO_2 _due to decreased cardiac output [9], and/or true right-to-left (RL) shunting, which is classically considered to arise from the reopening of patent *foramen ovale *[3,10,11]. In clinical practice guidelines for the diagnostic process of PAH, transthoracic contrast echocardiography is recommended to look for evidence of intracardiac shunting [11]. However, little evidence is available in the medical literature regarding this issue.

Here, we studied consecutive patients with precapillary PH, hypoxemia, and true RL shunting, in whom transthoracic contrast echocardiography was available, to determine whether true RL shunting causing hypoxemia is due to intracardiac shunting and especially patent *foramen ovale*.

## Methods

A computer-aided search was conducted to identify all adult patients evaluated for precapillary PH at our institution between January 2001 and March 2007. Patients with RL shunting and available transthoracic contrast echocardiography were then selected using the computerized database of the department of pulmonary function tests and manual review of the medical records, respectively. The study was approved by the Institutional Review Board of the *Société de Pneumologie de Langue Française*. Informed consent was obtained.

Inclusion criteria for this study included: (1) precapillary PH as defined by mean pulmonary arterial pressure (mPAP) greater than 25 mmHg at rest, with PAWP 15 mmHg or less, and PVR greater than 240 dyn.s.cm^-5 ^at right heart catheterization; [11] (2) hypoxemia defined by PaO_2 _at rest less than 10 kPa; (3) RL shunting as defined by shunt fraction (Qs/Qt) greater than 5%; (4) elevated AaPO_2 _under 100% O_2_; and (5) transthoracic contrast echocardiography performed within 3 months.

Causes of PH were classified according to the 2003 Venice classification [1]. Patients with PH associated with congenital systemic-to-pulmonary shunts and those with a known comorbidity potentially associated with a RL shunt (e.g. hemorrhagic hereditary telangiectasia) were excluded.

Shunt ratio (Qs/Qt) was calculated with the formula (Cc - Ca)/(Cc - Cv) while breathing 100% oxygen [12]. Qs and Qt corresponded to the shunt output and to total blood flow through the lungs, respectively. Ca and Cv corresponded to the oxygen contents of arterial and venous blood, respectively. Cc was the oxygen contents of end-capillary blood (with capillary PO_2 _estimated as similar to alveolar PO_2_). In 10 patients in whom Cc was not available, Qs/Qt was estimated with the formula (PAO_2 _- PaO_2 _mmHg)/(PAO_2 _- PaO_2 _mmHg + 1670) under 100% oxygen [13]; estimation of Qs/Qt was validated in the remaining 24 patients by comparison of Qs/Qt and estimated Qs/Qt using Bland-Altman method; the bias value was -0.1% ± 0.67 (95% confidence interval, -1.46 – 1.16).

Pulmonary function tests were performed according to the European Respiratory Society guidelines [14]. AaPO_2 _while breathing 100% O_2 _was performed as described elsewhere [15]; briefly, PaO_2 _was measured while the patient had been breathing 100% O_2 _for at least 10–15 minutes, with a deep inspiration every minute and immediately before drawing blood. Blood gas measurement was performed immediately after blood drawing, in the same room, with particular attention to eliminate air bubbles in the syringes. The actual P_I_O_2 _was measured to estimate PAO_2_, with PAO_2 _(kPa) = P_I_O_2 _- 6.27 - (PaCO_2_/0.8), and AaPO_2 _= PAO_2 _- PaO_2_. Thresholds of AaPO_2 _(18.6 kPa in supine position, and 24.5 kPa in upright position) determined previously [15] have excellent specificity (98%), positive predictive value (97%), and positive likelihood ratio (21.8) for the diagnosis of RL shunting higher than physiological shunting (5%).

Transthoracic contrast echocardiography (second harmonic imaging) was performed by injecting 10 ml of agitated isotonic saline solution mixed with 0.5 ml of room air into a humeral vein while simultaneously imaging the atria from the apical 4-chamber view with 2-D echocardiography for at least 12 cardiac cycles. Contrast echocardiography was performed both in supine and sitting position, with Valsalva manoeuvre when negative. Intracardiac RL shunt was defined by appearance of any contrast in the left atrium within 4 cardiac cycles, and intrapulmonary shunt by a delayed contrast appearance (more than 5 cardiac cycles).

Right heart catheterization was performed using standard procedures. A Swan-Ganz catheter was introduced into an antecubital or femoral vein and guided under radioscopic control into the pulmonary artery, until wedged position was reached. Pressures were measured according to standard procedures. Cardiac output was calculated by cold thermodilution (Edwards Lifesciences, Germany) from at least 5 measurements. Arterial blood was drawn from the Swan-Ganz catheter for the measurement of PO_2 _and the mixed venous oxygen saturation (SvO_2_) (ABL820 Radiometer, Copenhagen). Acute vasodilatator response was defined by a reduction of mPAP ≥ 10 mmHg to reach an absolute value of mPAP ≤ 40 mmHg, with an increased or unchanged cardiac output [16,17].

Microsoft Excel 2003 and SPSS 12.1 were used for data analysis. Data were presented as mean ± SD (range). Comparisons between groups were performed using the Mann-Whitney U test, Kruskal Wallis test, and linear regression analysis, when appropriate. Comparison of distribution of causes were performed using Chi-square analysis. Two-tailed p values < 0.05 were considered statistically significant.

## Results

### Study population

**Figure 1:**
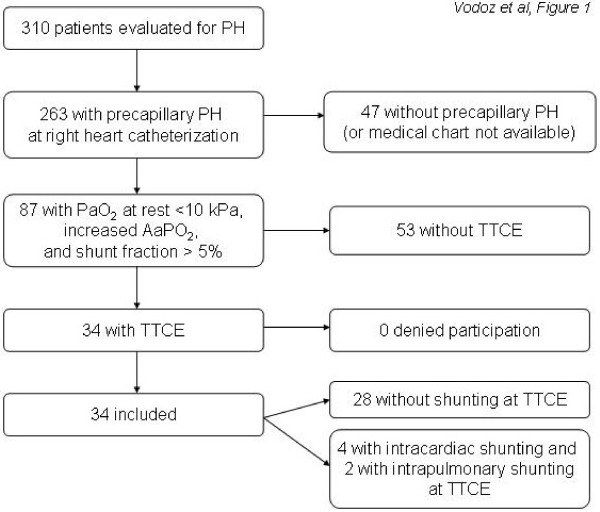
**Flowchart summarizing the selection and inclusion process**. PH, pulmonary hypertension; TTCE, transthoracic contrast echocardiography.

Among 310 patients evaluated in our department for PH during the study period, 263 had precapillary PH at right heart catheterization; 34 of them fulfilling the other inclusion criteria were included (figure 1). The clinical characteristics of the patients are shown in table 1. The mean age was 64 ± 15 years, and the male/female sex ratio was 1.3.

**Table 1:** Clinical and functional characteristics at the time of diagnosis of pulmonary hypertension.

*Clinical and functional parameters*	*Mean ± SD (range)*
Age, yr	64.0 ± 15 (25–82)
Sex (M/F)	19/15
Ex- or active smokers, %	50
Dyspnea NYHA class III–IV, %	76
6-min-walk distance, m	271 ± 116 (32–465)
TLC, % predicted	89 ± 16 (51–121)
FEV_1_, % predicted	76 ± 20 (33–117)
FEV_1_/FVC, %	65 ± 11 (36–88)
DLco, % predicted	49 ± 30 (12–113)
DLco/VA, % predicted	51 ± 28 (11–113)
PaO2, kPa	6.8 ± 1.3 (4.5–10.0)

FEV1, forced expiratory volume in one second; TLC, total lung capacity; DLco, single-breath diffusing capacity of the lung for carbon monoxide; VA, alveolar volume; NYHA, New York Heart Association.

Data expressed are mean ± SD (range) for quantitative values.

Etiologic groups of PH were as follows (table 2): 21% with PAH (etiologic group 1); 47% with PH associated with chronic parenchymal lung disease (group 3); 26% with PH related to thromboembolic obstruction of distal pulmonary arteries but not amenable to surgery (group 4); 6% with other causes (group 5). Distribution of etiologic groups did not differ from the overall population of 263 patients evaluated for precapillary PH in our department.

**Table 2:** Etiologic groups of pulmonary hypertension [1].

*Etiologic group*	
Pulmonary arterial hypertension (group 1)	*7 (21%)*
Idiopathic	6 (18%)
Portal hypertension	1 (3%)
Associated with lung disease and/or hypoxemia (group 3)	*16 (47%)*
Chronic obstructive pulmonary disease	8 (23%)
Interstitial lung disease	3 (9%)
Combined pulmonary fibrosis and emphysema	3 (9%)
Other	2 (6%)
Thromboembolic obstruction (group 4)	*9 (26%)*
Miscellaneous (group 5)	*2 (6%)*

Data expressed are absolute values (percent of total).

Most patients had severe dyspnea, as the median New York Heart Association (NYHA) functional class was III, and 76% of patients had functional class III–IV. Accordingly, the mean distance at 6-minute walk test was 271 ± 116 m, with a mean desaturation of 12 ± 7% at pulse oximetry, despite nasal oxygen during the test (4.2 ± 3.7 L/min of oxygen). The diffusion capacity of the lung for carbon monoxide was markedly decreased (49 ± 30%), whereas the mean value of total lung capacity was 89 ± 16% of predicted, and forced expiratory volume in one second/forced vital capacity was 65 ± 11% (table 1).

Mean mPAP (at rest in supine position and while breathing room air) was 45.8 ± 10.8 mmHg (table 3), with mPAP higher than 35 mmHg in 88% of patients. Mean PVR was 469 ± 275 dyn.s.cm^-5^. Mean cardiac index was 2.2 ± 0.6 L/min/m^2^. Acute vasodilatator response tested with inhaled nitric oxide was present in none.

**Table 3:** Hemodynamic characteristics at the time of diagnosis of pulmonary hypertension.

*Hemodynamic parameters*	*mean ± SD (range)*
RAP, mmHg	8 ± 4 (2–16)
mPAP, mmHg	46 ± 11 (30–68)
PAWP, mmHg	8 ± 3 (2–14)
Cardiac index, L/min/m^2^	2.2 ± 0.6 (1–3.4)
SvO2, %	55.8 ± 8.5 (41–72)
PVR, dyn.s.cm-5	469 ± 275 (142–1183)
PVRI, dyn.s.cm-5/m^2^	819 ± 446 (262–2082)

PAWP = pulmonary arterial wedge pressure; mPAP = mean pulmonary arterial pressure; RAP = right atrial pressure; PVR = pulmonary vascular resistance; PVRI = pulmonary vascular resistance index; SvO2 = venous oxygen saturation.

### Right-to-left shunting

According to inclusion criteria, hypoxemia was present in all patients, with a mean PaO_2 _of 6.8 ± 1.3 kPa (4.5–10.0) in room air in supine position, and 6.8 ± 1.4 kPa (4.9–10.2) in upright position. True RL shunting was present in all patients, with a mean Qs/Qt of 10.2 ± 4.2%. AaPO_2 _while breathing 100% oxygen was 32.5 ± 12.4 kPa (17.3–62.5) in supine position, and 27.5 ± 14.4 kPa (4.5–75.5) in upright position.

**Figure 2:**
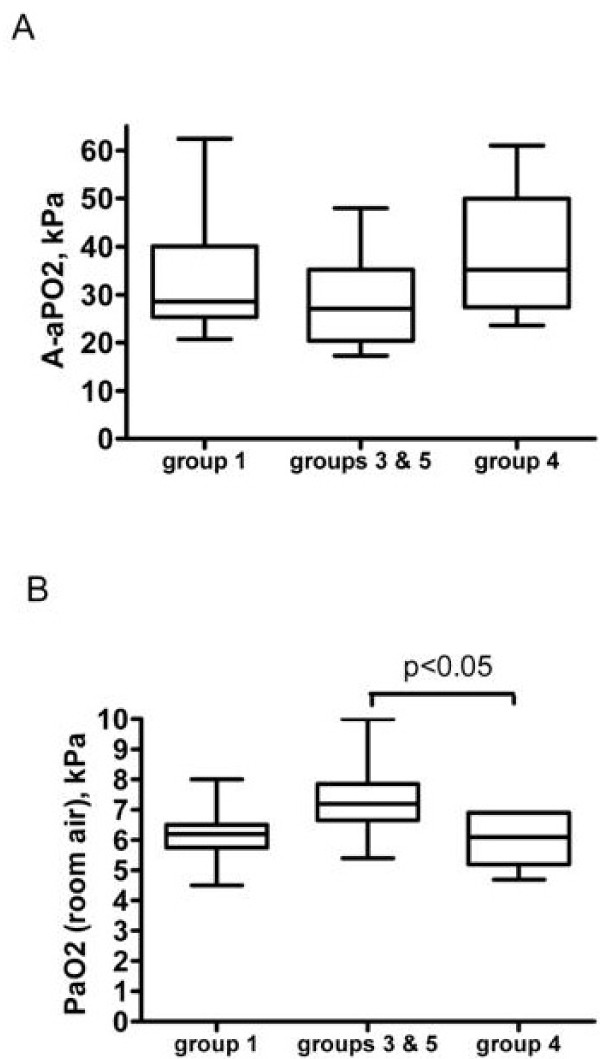
**A, Measurement of AaPO2 while breathing 100% oxygen according to the etiologic groups of patients (difference between groups was not significant by oneway analysis of variance)**. B, PaO_2 _on room air according to the etiologic groups (difference between groups was significant by Kruskal Wallis analysis, with p < 0.05; two-by-two post-hoc analysis was significant between groups 3 and 5, and group 4 by Bonferroni's multiple comparison test, p < 0.05). Group 1, pulmonary arterial hypertension; group 3, pulmonary hypertension associated with pulmonary diseases; group 4, chronic thromboembolic pulmonary hypertension; group 5, miscellaneous.

The value of AaPO_2 _was not significantly different between etiologic groups of precapillary PH (Kruskal-Wallis analysis, p > 0.05) (figure 2), although PaO_2 _differed significantly between etiologic groups of PH (p < 0.05), with higher PaO_2 _at room air in patients with PH associated with pulmonary disease (etiologic groups 3 and 5) than in patients with chronic thromboembolic PH (group 4) (7.3 ± 1.3 versus 6.0 ± 0.8 kPa; p < 0.05 by Bonferroni's multiple comparison test). As expected, linear regression analysis showed that AaPO_2 _significantly correlated with Qs/Qt (r = 0.495; p < 0.05); however, no significant correlation was observed between AaPO_2 _(or Qs/Qt) and hemodynamic parameters or carbon monoxide diffusion capacity.

None of the patients had alveolar consolidation, dilatation of pulmonary vessels, or any other lung abnormality on chest CT that could have resulted in a RL shunting.

Transthoracic contrast echocardiography showed early or late positive contrast in only 6/34 patients (18%), including 4 patients (12%) with early contrast appearance demonstrating intracardiac RL shunting (3 patients with patent *foramen ovale*, and one patient with atrial septum defect), and 2 patients (6%) with delayed contrast appearance indicating presence of intrapulmonary RL shunting. Patients with positive contrast echocardiography included 2 patients with idiopathic PAH, 3 patients with PH associated with chronic obstructive pulmonary disease, and 1 patient with chronic thromboembolic disease. These 6 patients were followed for a mean of 27 months. In 2 patients, PH worsened despite therapy (epoprostenol and sildenafil; bosentan and sildenafil), AaPO2 remained elevated, and contrast echocardiography was unchanged; in three patients treated with epoprostenol and sildenafil, epoprostenol, and diuretics alone, PH was improved or stable, AaPO2 decreased to 10–17 mmHg, and follow-up contrast echocardiography performed in one patient with patent *foramen ovale *remained positive. One patient died one month after initial evaluation. Percutaneous occlusion of intracardiac RL shunting was not performed.

Clinical characteristics, pulmonary function tests, blood gas analysis at room air, echocardiography, chest imaging, and hemodynamic data were similar whatever the result of contrast echocardiography (data not shown); AaPO_2 _tended to be higher in patients with positive than in those with negative contrast echocardiography, although the difference was not statistically significant (38.3 ± 15.7 kPa versus 31.3 ± 11.2 kPa, respectively, Mann-Whitney U test, p = 0.27).

## Discussion

Here, we showed that, when present, true RL shunting is related to intracardiac shunt in only a minority of patients. Hence, intracardiac RL shunting (through reopening of a patent *foramen ovale *or atrial septal defect) was present in only 12% of the patients, despite a shunt fraction higher than physiological values (5%) and elevated AaPO_2 _in all patients. This frequency is comparable to that found in the general population [18,19], and in previous studies in PAH (18%) [5] or in precapillary PH (26%) [20]. Contrast echocardiography was performed by experienced operators involved in our previous studies on RL shunting [15,21]. Although transesophageal echocardiography was not performed, it is unlikely that important intracardiac shunt may have been missed by transthoracic contrast echocardiography, which has excellent sensitivity when the second harmonic mode is used [22,23]. Thus, we consider that RL shunting and elevated AaPO_2 _were not explained by intracardiac shunt in most patients.

Our findings are relevant for the clinical management of patients with precapillary PH, whatever the etiologic group. Because hypoxemia is a potent pulmonary vasoconstrictor, and can contribute to the progression of PH, it is recommended to maintain oxygen saturation at > 90% at all times [24]. However, hypoxemia related to RL shunting is poorly improved by supplemental oxygen therapy. Therefore, maintaining the adequacy of oxygenation, as recommended by clinical practice guidelines [24], may be difficult in patients with PH and true RL shunting. In addition, our results challenge the clinical utility of contrast echocardiography in patients with hypoxemia related to precapillary PH.

Our study included patients with most categories of the etiologic spectrum of precapillary PH, with no marked differences, thus showing that our findings are not restricted to any etiologic subgroup. Since the frequency and severity of RL shunting did not significantly differ between etiologic groups, and because no known cause of shunting other than PH was present, we consider that RL shunting was more likely related to precapillary PH than to the associated disease when present. It cannot be excluded that ventilation/perfusion mismatch participated to hypoxemia. Since Qs/Qt was greater than 5%, and AaPO_2 _did not correlate with cardiac output, it is unlikely that increased AaPO_2 _was due to low cardiac output. Interestingly, RL shunting (with increased AaPO_2 _and a median of Qs/Qt of 19%) was previously reported in 8 patients with severe PH associated with chronic obstructive pulmonary disease (with mPAP higher than 40 mmHg, "disproportionate" to the lung disease), with no evidence of intracardiac shunting at contrast echocardiography [25]. PH was moderate or severe in 88% of our cases. Whether treatment of PH affects RL shunting and hypoxemia remains to be determined, however some improvement of AaPO_2 _was observed with treatment of PH in few patients.

The pathophysiology of RL shunting and increased AaPO_2 _in our patients remains largely unknown. RL shunting was higher than physiological shunting, which represents less than 5% of cardiac output [26]. Transthoracic contrast echocardiography reportedly has excellent sensitivity for the detection of intrapulmonary shunt [27]. Experimental studies in normal humans and dogs have shown increased RL shunting at exertion demonstrated by elevated AaPO_2_, positive transthoracic contrast echocardiography, and isotope-labeled microspheres, in proportion to the increase of cardiac output [28-30], especially under hypoxic conditions [31], although with unclear consequences on PaO_2_. Studies in infants [32] and adults [33] have demonstrated intrapulmonary arteriovenous shunts, with up to 200 μm in diameter. Large-diameter (> 25 μm) intrapulmonary arteriovenous pathways may be recruited with physiological exercise [30], thereby limiting the rise in PAP despite cardiac output increase [34]. In patients with PAH, dilated and distorted capillary circulation were assumed to reflect collateral flow around obliterated pulmonary arterial segments [35]. Intrapulmonary shunting in PH may be regulated by pulmonary vascular pressure and flow [29], and may take place at the capillary level, the diameter of which may be higher than normal (7–11 μm) but small enough to prevent the transit of microbubbles (60–90 μm). RL shunting may further increase at exercise in patients with PH [36]. Thus, RL shunting in patients with PH might represent shunting through intrapulmonary arteriovenous pathways recruited with increase in microvascular pressure, similar to mechanisms seen during physiological exercise [30,31] or hepatopulmonary syndrome [37]. Alternatively, it might be due to increase in complex anatomic anastomosis of bronchial and/or pleural circulation with the pulmonary circulation, as suggested in PH [38] and especially chronic thromboembolic PH [39].

Our study had limitations, including its retrospective design, and heterogeneity of causes of PH with potential selection bias. Although patients with various causes of precapillary PH were included, the distribution of causes of PH was similar in patients with or without RL shunting within the overall population of patients with PH in our center, and we consider that similar results would have been obtained had the patient population been restricted to PAH. We could not determine whether occurrence of RL shunting was related to more severe hemodynamic parameters, although RL shunting was observed mostly in patients with moderate to severe PH. The effect of exercise on RL shunting was not evaluated. Contrast echocardiography was not performed in normoxic patients.

## Conclusion

RL shunting was not related to reopening of patent *foramen ovale *in most patients with precapillary PH and hypoxemia related to RL shunting, as opposed to classical concepts. Our findings need confirmation by prospective systematic evaluation of hypoxemia, shunt fraction, AaPO_2_, and transthoracic and/or transesophageal echocardiography in consecutive patients with precapillary PH. Physiological studies are strongly needed to determine the mechanism of RL shunting and hopefully to contribute to better management of patients with PH and hypoxemia.

## Competing interests

The authors declare that they have no competing interests.

## Authors' contributions

JFV and VC performed the analysis and wrote the article. GD analysed echocardiography data. CK, ASB, BM, and JFM analysed clinical data. JYB and JCG performed the pulmonary function tests. JFC designed the study and contributed to the analysis of data and writing of the manuscript. All authors have read and approved the final manuscript.

## Pre-publication history

The pre-publication history for this paper can be accessed here:


